# Pharmacological Studies of Medicinal Potential Phytochemicals in Plant Roots

**DOI:** 10.3390/ph16111520

**Published:** 2023-10-26

**Authors:** Monika Stompor-Gorący

**Affiliations:** Department of Human Pathophysiology, Institute of Medical Sciences, Medical College of Rzeszów University, Kopisto 2a, 35-310 Rzeszów, Poland; monika.stompor@gmail.com

Despite various limitations, there is a growing interest in the development of phytomedicine as an alternative therapeutic method, which uses herbal preparations exerting a positive effect on the human immune system and supporting conventional therapies [1,2]. Currently, the main trend in this branch of science is to discover new formulations, innovative molecular structures and derivatives of known substances found in various parts of plants, and to find their new applications [3,4,5,6]. In vitro and in vivo pre-clinical trials have shown that herbal drugs can interact with other drugs, such as chemotherapeutics used for cancer treatment, increasing their therapeutic efficacy [7,8]. Nevertheless, both single compounds and multicomponent formulations, except for their therapeutic effects, may also have adverse effects. Therefore, it is of crucial importance to determine in detail the chemical composition of plant extracts, analyse their pharmacological properties and their effects on the human body [9,10]. Thus, a paramount goal of phytochemical analyses is to standardize physicochemical and analytical techniques that are used for the development of safe and efficacious herbal formulations with therapeutic potential [11].

The objective of this Special Issue is to summarize and disseminate the most recent advances in the research concerning phytochemical analysis of plant roots, with special attention to bioactive compounds with health-promoting and therapeutic properties.

This Special Issue fills the gaps in the current knowledge on the therapeutic potential of phytocompounds that are found in plant roots. In five published original scientific papers, attention has been paid to various aspects of the treatment of diseases, such as chronic kidney disease and bacterial infections, but also a range of biological activities of bioactive root compounds have been studied, including their toxicity on neurobehavioral and reproductive function and inflammatory activity [12,13,14,15,16].

The immunomodulatory effect of an aqueous extract of black radish (*Raphanus sativus* var. *niger* (Mill.) J.Kern.) consisting of novel water-soluble polysaccharides was confirmed using the RAW 264.7 mouse macrophage cell line and also mouse peritoneal macrophages. The root hot water extract increased the expression of pro-inflammatory cytokines (IL-1β, IL-6, and TNF-α), iNOS, and COX-2 as well as induces macrophage activation through the TLR2/4–MAPK–NFκB–Akt–STAT3 signaling pathway [12]. In turn, Acero et al. [13] described the phytochemical composition and antiinflammatory and antioxidant action of the rhizome hexane extracts obtained from *Acanthus mollis* L. The authors stated that its antiinflammatory capacity was mediated by a decrease in NO production and by the prevention of damage caused by oxidative stress, both through the scavenging of ROS and through the antioxidant cellular enzyme system. In the next paper, the deleterious effects of pirimicarb—one of the carbamate insecticides employed as an aphidicide—on the brain, behavior, and reproductive system of male albino rats was elucidated. It was documented that *Ephedra alata* subsp. *monjauzeana* Dubuis & Faurel crude extract, commonly used as herbal tea, can prevent destructive neurological and reproductive manifestations caused by the tested primor [14]. Furthermore, the antimicrobial compounds against multidrug-resistant skin-related pathogens (like e.g., *S. aures*, *S. saprophyticus*, *S. epidermidis*, *S. haemolyticus*) were identified in *Asphodelus bento-rainhae* subsp. *bento-rainhae* and *Asphodelus macrocarpus* subsp. *macrocarpus* root tubers with 70% ethanol extract by Malmir et al. [15]. The renoprotective effect of *Chrysanthemum coronarium* L. (*Glebosis coronaria* (L.) Cass ex. Spach) extract on improving renal function and adenine-induced chronic kidney disease in mice was confirmed by the Kim et al.’s study [16].

Moreover, this Special Issue contains a review of the health-promoting properties and most current detection methods of baicalin (7-D-glucuronic acid-5,6-dihydroxyflavone)—a natural flavonoid extracted from the roots of *Scutellaria baicalensis* Georgi [17].

We encourage researchers to draw inspiration from this Special Issue to develop new methods of prevention and treatment of various diseases with the help of alternative, non-invasive and readily available phytomedicine.
